# Investigation of the 1-week effect of traffic light nutrition labeling on diet selection among Japanese university students: a randomized controlled trial

**DOI:** 10.1186/s12889-024-17815-4

**Published:** 2024-02-05

**Authors:** Nobuyuki Wakui, Raini Matsuoka, Kotoha Ichikawa, Chikako Togawa, Aika Okami, Shoko Kawakubo, Hinako Kagi, Mai Watanabe, Yuika Tsubota, Miho Yamamura, Yoshiaki Machida

**Affiliations:** Division of Applied Pharmaceutical Education and Research, Faculty of Pharmaceutical Sciences, Hoshi University, 2-4-41 Ebara, Shinagawa-ku, 142-8501 Tokyo, Japan

**Keywords:** Healthy dietary choices, Traffic light food label, Front of Pack, Nutrition label, Food labeling system, Nutritional content

## Abstract

**Background:**

The method of displaying nutrition information labels on the front of food packaging (FOP: Front of Pack) has been implemented worldwide to prevent lifestyle-related diseases. This study aimed to investigate whether the use of the UK’s Traffic Light Food (TLF) label, known as the FOP label, influences the dietary choices of Japanese youth and promotes healthy dietary choices.

**Methods:**

Diet selection was performed for one week each during the baseline and intervention periods. During the intervention period, TLF labels were displayed on meal images of the intervention group. Participants chose what they would like to have for dinner of the day from 15 images. Each meal was scored based on the color of the nutrition label, and a comparison between groups was made to determine whether TLF labeling influenced meal selection for dinner. The psychological stress caused by the presence or absence of nutrition labels and nutritional components when choosing meals was also evaluated.

**Results:**

A total of 69 participants were randomly assigned to two groups. Dietary choice scores indicated that the TLF-labeled group made significantly healthier dietary choices than the unlabeled group. Additionally, the TLF-labeled group showed a significant increase in the percentage of people conscious of nutritional components when choosing meals. Furthermore, a significant increase in the number of people conscious of protein, a nutritional ingredient not indicated on the TLF label, was observed. During the test period, no difference in psychological stress caused by the presence and absence of the TLF labels was observed.

**Conclusions:**

The use of TLF labels also encouraged healthy dietary choices among Japanese university students. The use of FOP nutrition labels should be considered in Japan to prevent lifestyle-related diseases through healthy dietary choices.

**Trial registration:**

UMIN Clinical Trials Registry Number: UMIN000047268. Registered March 23, 2022.

## Background

The rise in lifestyle-related diseases has become a global concern. Particularly, the increase in the number of obese people has become a serious issue [1–4]. In 2025, one in five of the world’s population will become obese if effective measures are not taken [1, 3]. Obesity has been reported to be associated with many chronic diseases, such as diabetes and heart disease [2, 5, 6]. Obesity has led to an increase in critically ill patients in the community [7], greatly decreased life expectancy [8], and even become a threatening factor for healthcare [5]. Thus, the global spread of obesity prevention has become one of the important public health efforts [9–11].

Methods for labeling nutritional information at the front (FOP: Front of Pack) of food packaging have been widely adopted worldwide [12]. This mechanism can help consumers voluntarily select healthy foods during food purchases [13] and is currently considered for introduction worldwide [14–21]. Specifically, the guideline daily amount (GDA) system, which displays a ratio to the daily intake reference [22], the color-coded GDA system, which also uses color to indicate whether it is appropriate against GDA reference [12], the summarized system, which displays a global health index for foods based on calculated scores [23–25], and the nutritional alert system, which expresses a high content of certain nutrients [26, 27], are famous and used systems.

The United Kingdom is the first European country to introduce FOP, and the signal machine food (TLF: Traffic Light Food) labeling of the color-coded GDA system developed in the country is known worldwide [12, 28]. This TLF labeling has spread to many countries outside the United Kingdom [29, 30], as healthy eating can be easily judged by color. Incidentally, labeling has been shown to contribute to improving health awareness, and it can also be expected to be effective in preventing lifestyle-related diseases [31–34].

The use of TLF labeling (red, yellow, and green) allows consumers to select a healthy diet during food purchases. The color-coded FOP labeling system is used to indicate the nutritional quality of a food based on four components, including total lipids, saturated fatty acids, sugars, and salts [35, 36], each of which is labeled in red if it is present in excessive amounts in the food, green if appropriate, and yellow if intermediate. Since it is easy to notice visually and can be judged at a glance, it will increase health consciousness when purchasing food. Thus, it can be expected to prevent lifestyle-related diseases such as obesity. Additionally, its usefulness has been reported worldwide [29, 30].

In Japan, the new food labeling system was fully enforced on April 1, 2020, against the background of increasing health awareness, increasing the importance of nutrition labeling, and expanding mandatory nutrition labeling abroad. Thus, nutritional labeling of foods became mandatory [37], labeling of energy, proteins, fats, carbohydrates, and salts became required, and labeling of saturated fats and dietary fiber was also recommended [38]. However, because these labels are listed on the backside of food packages, they are difficult to use when consumers are unaware of nutrition in their daily lives. Additionally, unlike TLF labeling, which provides visual nutritional information, it is difficult for the public to effectively use it as in other countries because it is necessary to determine the appropriate nutritional content by themselves [39].

With the increasing number of obese individuals in Japan [40], there is a demand for more convenient and effective FOP labeling. Previous studies have evaluated the efficacy of TLF labeling among Japanese university students [41]; however, these studies focused only on the day following the diet selection. The continuous use of TLF labeling over the course of one week and its potential variations in food choices between weekdays and weekends have not been explored. Internationally, long-term studies on TLF are mostly observational [42] or focus on the influence of label colors [43], not on sustained behavioral change.

Therefore, this study aimed to verify the usefulness of the continuous use of TLF labels by the introduction of TLF labels to indicate nutrients on the front of food packaging for Japanese university students. This represents the first intervention study to scrutinize the week-long impact of Front of Pack Food Labels (FOPL) in this demographic, aiming to provide insights into their role in fostering consistent healthy eating habits.

## Methods

### Study plan and setting

This was an interventional trial conducted on university students between April 11 and May 2, 2022. Data were collected through an online survey using a web-based questionnaire administered through Google Forms. Participants responded to questions on health- and nutrient-related awareness, stress, TLF labeling, and food choices.

### Study design

This was a randomized, double-blind, parallel-group study examining how nutritional labels influence university students’ dietary choices. For three weeks, participants selected their dinner from 15 displayed dietary images. In the first week, the images had no TLF nutrition labels. After a 1-week washout, in the third week, the intervention group saw images with nutrition labels, while the non-intervention group viewed the same images without labels. The study assessed if the presence of nutrition labels made individuals more conscious of choosing healthy meals.

Written informed consent was obtained from all participants for participation in this study. The study protocol was approved by the Research Ethics Committee of the Hoshi Pharmacy University (approval number: 2021-24) and pre-registered with the University Hospital Medical Information Network Center before study initiation (UMIN study ID: UMIN000047268, first registered 23/03/2022). All procedures performed in this study involving human participants were conducted in compliance with relevant guidelines, including the Helsinki declaration and its later amendments or comparable ethical standards.

### Participants

Inclusion criteria included university students who gave consent for participation in the study and males and females aged 18 years or older at the time of informed consent. Exclusion criteria included university students who refused to participate in the study due to their intention, those with dietary restriction due to metabolic and endocrine diseases, those with eating disorders, those with anorexia, those with intentional or unintentional weight gain or loss of more than 5% in the past 3 months, and those who were judged by the clinical trialists to be inappropriate for inclusion in the study. We conducted random recruitment of participants among students from Hoshi University.

### Randomization and masking

Participants were allocated in a 1:1 ratio into two groups. Random allocation was conducted by a third party using permuted block methods with block sizes of 2 and 4. The results of this allocation were securely stored in a dedicated vault with a locked key to ensure blinding. Following this, a food choice survey was conducted in a double-blind manner. Participants received the URL of the Google Forms via email, either with TLF labels or without, based on their group assignment. They were instructed to answer the survey independently to avoid discussing its content with others. The blinding of both intervention implementers and data analysts was upheld until the completion of the statistical analysis.

### Procedure

The study was conducted using Google Forms without contact between the researchers and the participants. A URL of Google Forms of the food choice survey was transmitted simultaneously to all participants at 15 o’clock on the day of the survey. Participants answered the survey between 15 and 20 o’clock in 30 min (before dinner). The answers were made at least 2 h after lunch and were performed by avoiding when the stomach was full. Real-time data entry monitoring was conducted online to identify whether participants’ responses were performed properly every day.

For the intervention, the group was presented with meal images displaying TLF labels and asked to select what they wanted to eat for dinner that day. The nutritional content displayed on these labels—total fat, saturated fat, sugar, and salt—was in accordance with the UK Food Standards Agency’s guidelines [12]. The meals available for selection included 15 types of Japanese bentos as described in Wakui et al. [41], which were color-coded based on their popularity derived from an always better control (ABC) analysis of participants’ preferences collected prior to the intervention.

In this analysis, meals with higher popularity were labeled with a red tag, less popular ones with yellow, and the least popular with blue. It should be noted that the red, yellow, and blue colors on our TLF labels did not correspond to the actual nutritional values of the meals. Rather, they served as indicators in a behavioral experiment designed to determine whether the colors associated with the TLF labels would influence participants’ meal choices. Traditionally, traffic light labels use red, yellow, and green; however, in our study, we used blue instead of green for the ‘healthier’ choices. This choice was informed by the cultural context in Japan, where the ‘go’ signal in traffic lights is perceived as blue. By labeling the less popular foods with blue, we aimed to align with this cultural perception, thus facilitating a more intuitive selection process for the Japanese participants. This method of color coding was employed as a way to explore if TLF could influence participants’ selection towards these less popular, and potentially healthier, choices.

The nutritional values displayed on the labels were determined by randomly generating numbers within the nutrient range specified by the UK Food Standards Agency (FSA) guidelines for each color category assigned through the ABC analysis. This means that for meals labeled red, the nutritional values were randomly generated to fall within the range with higher levels of FSA’s ‘red’ criteria. Similarly, for meals labeled blue, the values corresponded to the ‘healthier’ range of the FSA’s criteria. This approach ensured that the labeling was consistent with recognized standards while allowing us to investigate the influence of color-coded TLF labels on food selection behavior within the experimental context.

Additionally, we did not uniformly color-code all nutritional components to ensure that the labels mirrored the complexity and variety found in real-world food packaging. This decision was also in line with the intent to reflect the dietary attentiveness of Japanese individuals in their twenties to specific nutrients [44]. Total fat and salt, being nutrients monitored by this age group, were colored based on their ranking in the ABC analysis. Conversely, saturated fats and sugars were assigned colors of blue or yellow randomly. This strategy prevented a monochromatic appearance of nutritional labels, thereby maintaining the realistic and varied look that consumers are used to.

### TLF labeling color setting

Our methodology for creating the TLF labels was carried out in two primary steps. The first step involved using the ABC analysis to assign colors based on the popularity of meals among participants: red for the most popular, yellow for moderately popular, and blue for the least popular meals. The second step entailed assigning nutritional values within defined ranges according to the UK Food Standards Agency’s guidelines, correlating with the color coding from the first step. This two-step approach allowed us to create a label system that both reflects the cultural context of our Japanese study population and adheres to internationally recognized nutritional standards.

**Fig. 1:**
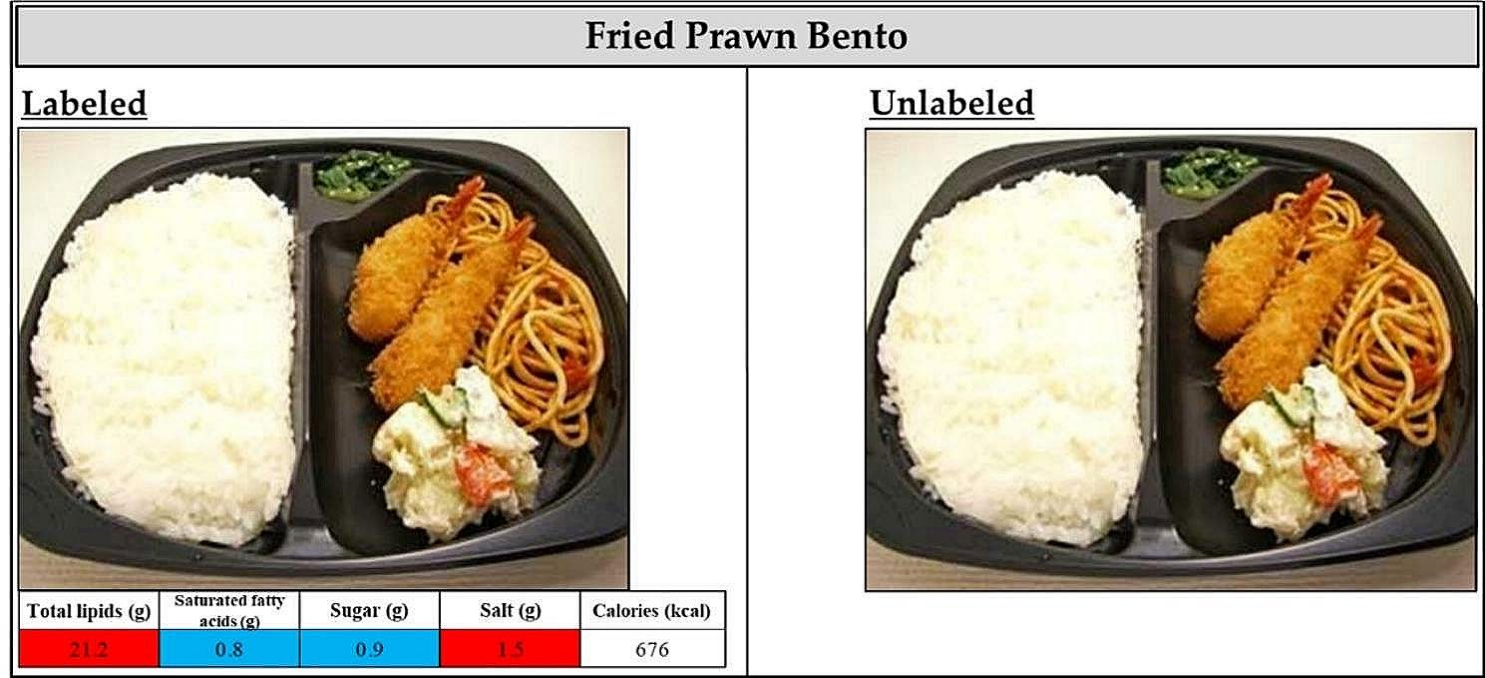
Traffic light labeling (TLF) system example on a study meal. Participants were divided into two groups during the intervention period, one with labels and the other without, and selected their desired meal for dinner from fifteen different types of meals

According to the UK Food Standards Administration guidelines, the color setting criteria for nutrition labeling in this study were as follows [12]: For total fat labeling, diets with total fat of > 17.5 g per 100 g were labeled in red, diets with total fat of ≥ 3.1 g and ≤ 17.5 g per 100 g were labeled in yellow, and diets with total fat of ≤ 3 g per 100 g were labeled in blue. For saturated fat labeling, diets with saturated fat of > 5 g per 100 g were labeled in red, diets with saturated fat of ≥ 1.6 g and ≤ 5 g per 100 g were labeled in yellow, and diets with saturated fat of ≤ 1.5 g per 100 g were labeled in blue. For sugar labeling, diets with sugar of > 22.5 g per 100 g were labeled in red, diets with sugar of ≥ 5.1 g and ≤ 22.5 g per 100 g were labeled in yellow, and diets with sugar of ≤ 5 g per 100 g were labeled in blue. Finally, for representation, diets with salinity of > 1.5 g per 100 g were labeled in red, diets with salinity of ≥ 0.31 g and ≤ 1.5 g per 100 g were labeled in yellow, and diets with salinity of ≤ 0.3 g per 100 g were labeled in blue. Figure 1 presents examples of the created meal labels and corresponding selection images.

### Outcomes

Outcomes are as follows;1) Whether the group with the TLF label has an increased percentage of people who consistently choose a healthy diet throughout the week (7 days from Monday to Sunday) than the group without the TLF label. 2) Whether the presence or absence of the TLF label makes a difference in the percentage of people who are conscious of healthy dietary intake and nutritional components such as calories, carbohydrates, and fats when making dietary choices. 3) Whether the use of the TLF label increases the stress caused by withholding what you want to eat.

And at the end of the study, all participants were asked whether the TLF label was valid.

### Statistical analysis

The demographic characteristics of the participants were determined using descriptive statistics. Numerical data were presented as mean and standard deviations. Categorical data were presented as frequency and ratio. Whether or not the primary endpoint, the presence or absence of TLF labels, influenced healthy eating choices at dinner was analyzed using analysis of covariance and a mixed-effects model with baseline values as covariates. For each color of the TLF label, the total score on the TLF label for 1 week by food selection was calculated as 3 points for the red label, 2 points for the yellow label, and 1 point for the blue label, and the differences in change were compared between groups from baseline using the obtained values. Whether the secondary endpoint of the presence or absence of TLF labels influenced the proportion of people making healthy eating choices at dinner and whether the proportion of people conscious of nutritional components differed during food choices according to the presence or absence of TLF labels were assessed using Fisher’s exact test. Additionally, the strength of impact by a nutrient composition by TLF labeling was assessed using Cramer’s V. Furthermore, whether TLF labeling affects stress using the stress rating scales—Depression Anxiety Stress Scale-21 (DASS-21) and Visual Analog Scale (VAS)—was assessed in a mixed-effects model with baseline values as covariates. Graphics were prepared using the ggplot2 package in the R statistical software (version 4.1.2, R Foundation for Statistical Computing, Vienna, Austria). Data analysis was performed using SAS (version 9.4, SAS Institute, Inc., Cary, NC, USA). The number of missing values was 0 because the Google Forms were prepared in such a way that the survey would not end even if only one part was left unanswered. A *p*-value of less than.05 was considered statistically significant.

## Results

### Participants’ characteristics

**Fig. 2:**
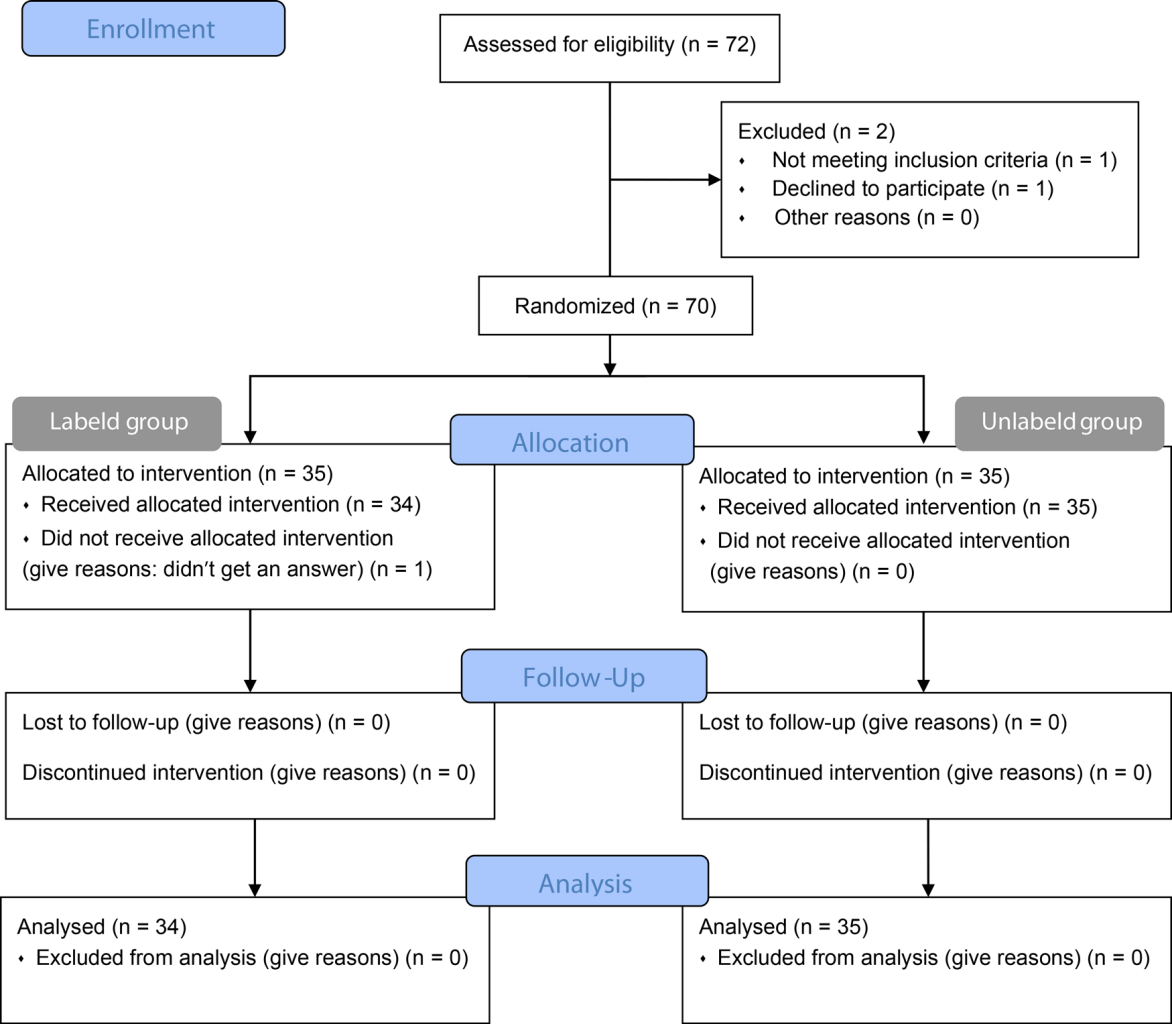
CONSORT flow diagram. Seventy participants were randomly allocated into two groups, each consisting of thirty-five individuals. In the final analysis, the labeled group comprised thirty-four members, while the non-labeled group retained all thirty-five

A total of 72 university students were enrolled. After applying the exclusion criteria, 70 students participated in this study. Participants were randomly assigned to either the TLF-labeled group (*n* = 35) or the TLF-unlabeled group (*n* = 35). Of the 70 participants, 97.1% (34/35) in the TLF-labeled group, 100.0% (35/35) in the TLF-unlabeled group, and 98.6% (69/70) in total completed the study (Fig. 2).

The demographics and baseline characteristics of the analysis population were similar between the two groups (Table 1). The overall mean age was 21.0 ± 1.3 years, and the majority of participants were female (92.8%). Appetite (Simplified Nutritional Appetite Questionnaire SNAQ) and stress (DASS-21 and VAS) were also similar between the groups. Less than 30% of individuals in both groups chose dinner with awareness of nutritional components at baseline. Also, the proportion of people aware of the nutritional content of calories, proteins, total lipids, sugar, saturated fatty acids, salts, and dietary fiber was small, and the proportion was similar between the two groups.

**Table 1:** Demographics and baseline characteristics (*n* = 69)

	Unlabeled group	Labeled group	p-value
	(*n* = 35)	(*n* = 34)	
Age	21.0 ± 1.2	21.0 ± 1.3	1.00
Sex (women)	33 (94.3%)	31 (91.2%)	0.67
SNAQ	15.83 ± 1.48	16.18 ± 1.47	0.33
TLF_SumScore	15.51 ± 1.96	15.50 ± 1.99	0.98
Nutritional consciousness when choosing meals			
Whole nutrition	8 (22.9%)	9 (26.5%)	0.79
Calories	4 (11.4%)	7 (20.6%)	0.34
Proteins	4 (11.4%)	8 (23.5%)	0.22
Total lipids	6 (17.1%)	7 (20.6%)	0.77
Sugar	1 (2.9%)	3 (8.8%)	0.36
Saturated fatty acids	1 (2.9%)	0 (0.0%)	1.00
Salt	0 (0.0%)	1 (2.9%)	0.49
Dietary fiber	6 (17.1%)	4 (11.8%)	0.73
Health awareness	9 (25.7%)	15 (44.1%)	0.13
DASS-21			
Total	5.11 ± 5.41	4.94 ± 5.48	0.90
Depression	1.66 ± 2.17	1.76 ± 2.36	0.84
Anxiety	0.91 ± 1.54	0.82 ± 1.53	0.81
Stress	2.54 ± 2.96	2.35 ± 2.96	0.79
VAS			
VAS1-1	6.54 ± 2.24	6.35 ± 2.06	0.72
VAS1-2	4.83 ± 2.63	4.70 ± 2.27	0.82
VAS1-3	6.43 ± 1.87	6.12 ± 2.09	0.53

SNAQ: Simplified Nutritional Appetite Questionnaire (used for Appetite Rating Scale); DASS-21: Depression Anxiety Stress Scale-21 (used for Stress Rating Scale); VAS: Visual Analog Scale (used for Stress Rating Scale). VAS1: Looking back over the past week, do you think you were able to lead a regular life? VAS2: How stressful did you look back in the last week? VAS3: Looking back over the past week, how positive have you been? TLF_SumScore: for each color of the TLF label, three points for red labels, two points for yellow labels, and one point for blue labels were used to calculate the total score on the TLF label by food preference during the baseline period

### Effect of the presence or absence of TLF label on dietary choices

**Fig. 3:**
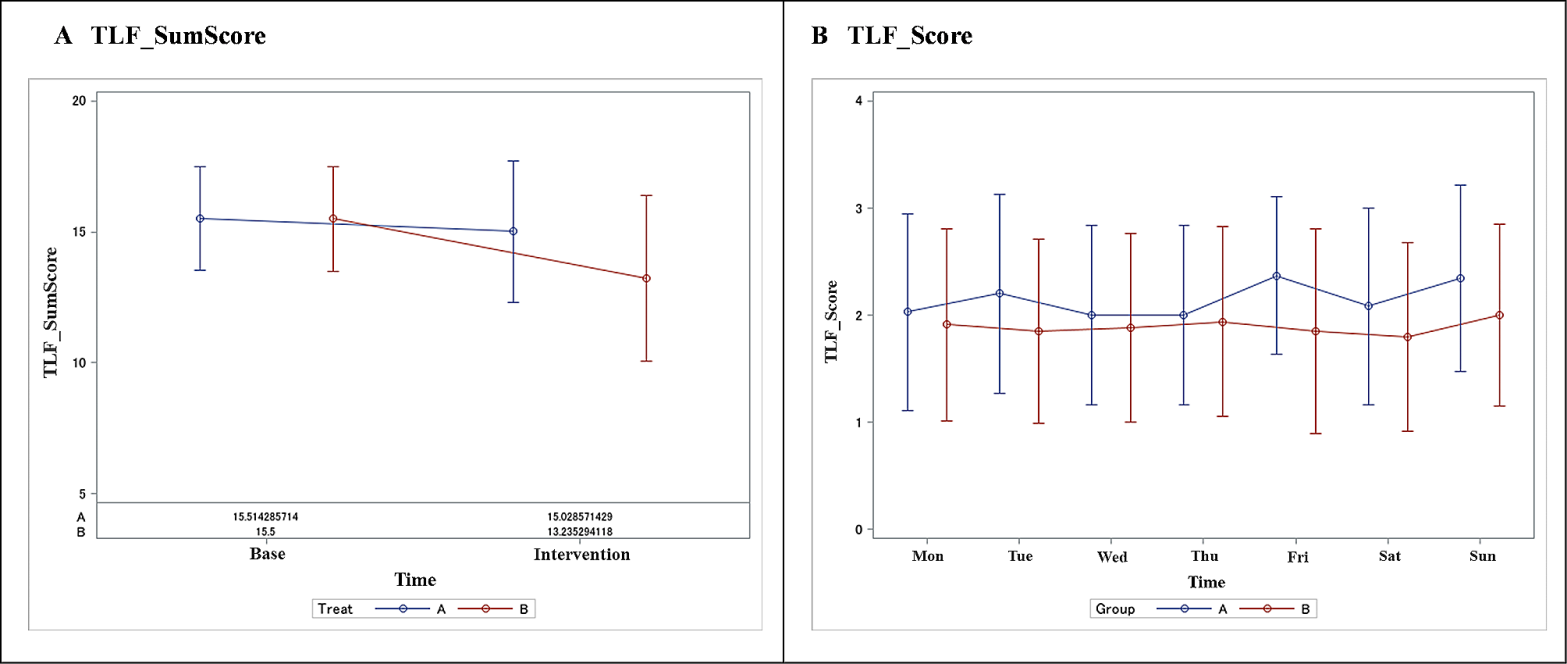
Changes in scores in dietary choices. (**A**) illustrates total scores during the baseline and intervention periods, while (**B**) breaks down scores by the day of the week during the intervention period. Across all days of the week, the labeled group consistently scored lower than the unlabeled group. TLF labels were color-coded, with red labels assigned 3 points, yellow labels assigned 2 points, and blue labels assigned 1 point. It’s important to note that the data in the figures represent graphical representations of raw data. Additionally, in (**B**), the scores remain relatively stable across different days of the week, showing a consistent horizontal trend

Figure 3 shows changes in scores in 1-week dietary choices. The average total score during the baseline period in the unlabeled group was 15.5 ± 2.0 points and during the intervention period was 15.5 ± 2.0 points, showing no difference in the scores between the two groups. However, the mean total score during the intervention period in the labeled group was 13.2 ± 2.0 points and in the unlabeled group was 15.5 ± 2.0 points, indicating a decrease in the total score in the labeled group.

Figure 3A shows the total score during the baseline and intervention periods. Figure 3B shows the scores by day of the week for the interventional period. Overall, the scores were lower on all days of the week for the labeled group than for the unlabeled group. Group A is the unlabeled group, and Group B is the labeled group. Each color of the TLF label was scored as 3 points for the red label, 2 points for the yellow label, and 1 point for the blue label.

Table 2 shows the change in the sum score during the 1-week dietary selection for each group. The change from baseline score was − 0.48 (− 1.39 to 0.42) in the unlabeled group and − 2.27 (− 3.19 to − 1.35) in the labeled group. The least squares mean difference (LSMD) was 1.78 (0.49–3.08), with a *p*-value of.008, which is significantly different.

**Table 2:** Amount of change in total scores for meal selection over the course of a week

Variable	Adjusted mean (95% CI)				
	Unlabeled group	Labeled group	LSMD	*p*-value	
Score change	−0.48 (− 1.39 to 0.42)	−2.27 (− 3.19 to − 1.35)	1.78 (0.49–3.08)	**0.008**	

LSMD: least squares mean difference. The decrease in score indicates that participants chose a healthier diet

### Change in the percentage of healthy dietary choices using TLF labels

**Fig. 4:**
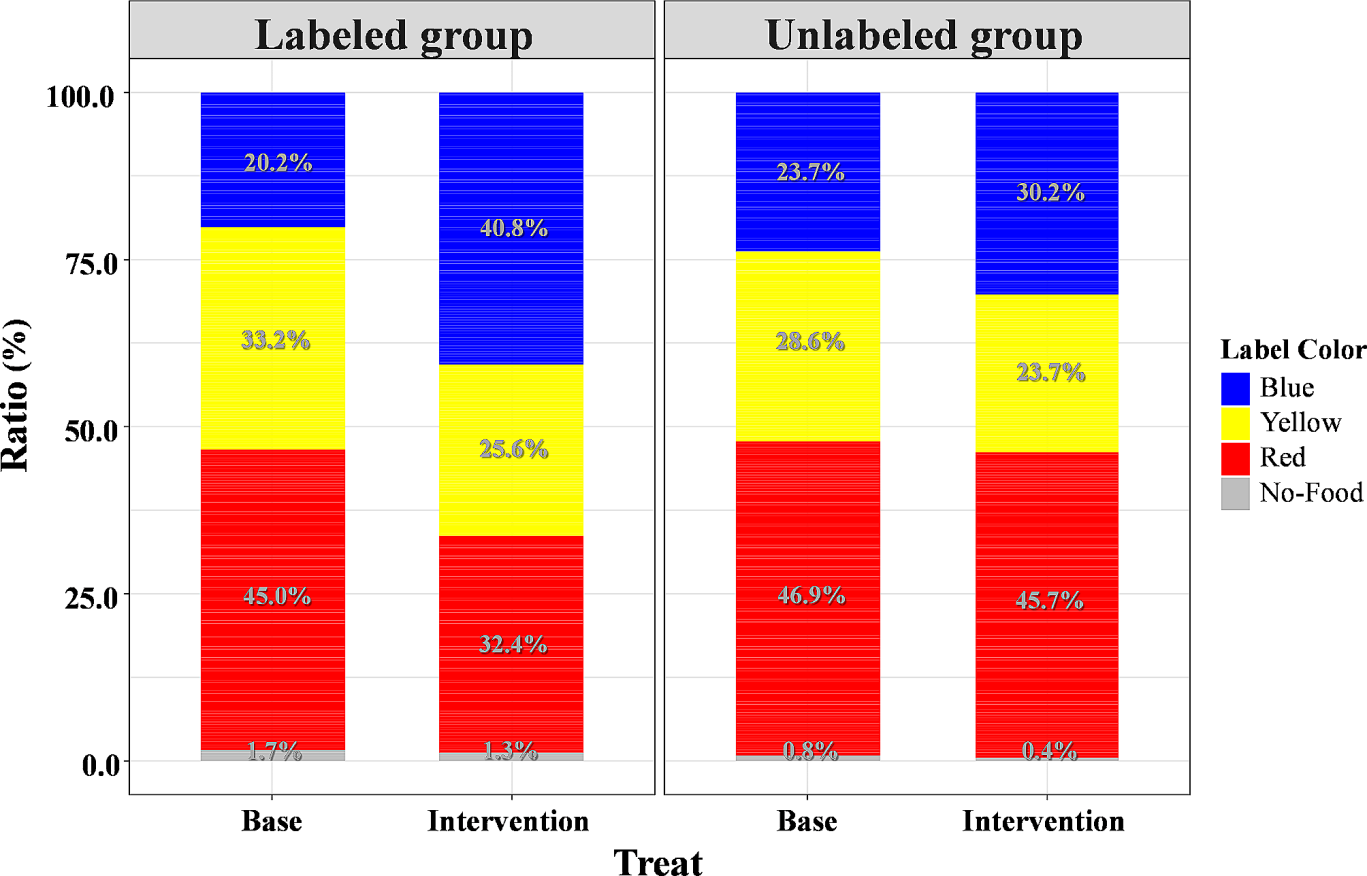
Change in proportion of healthy dietary choices before and after the intervention. Figure 4 displays changes in dietary choices between the baseline and intervention periods. In the labeled group, there was a 20.6% increase in blue label choices and a 12.6% decrease in red label choices during the intervention compared to the baseline. The unlabeled group had changes too, but the labeled group showed a 10.5% higher preference for blue labels and a 13.3% lower preference for red labels during the intervention. This difference was statistically significant (overall *p* =.011), indicating an increase in healthy food choices due to labeling

Figure 4 shows the percentages of dietary choices in the baseline and intervention periods. The aggregated results of the proportion of diets selected by participants in the red, yellow, and blue labels during the week of each period are shown in Fig. 4. In the comparison of each group before and after intervention, the proportion of dietary choices falling on the blue label increased by 20.6%, and the proportion of dietary choices falling on the red label decreased by 12.6% in the labeled group compared with the baseline period. However, the unlabeled group showed changes in dietary choices falling under the blue and yellow labels between the baseline and intervention periods. However, no change in the proportion of dietary choices falling on the red label was observed. In the comparison between groups during the intervention period, the proportion of dietary choices falling on the blue label was 10.5% higher, and the proportion of dietary choices falling on the red label was 13.3% lower in the labeled group than in the unlabeled group. Furthermore, a significant difference in the proportion of meals was observed in both groups (*p* =.011). From these two results, the percentage of people who choose to eat healthy due to labeling has increased was showed.

### Percentage of people who were aware of nutritional components during dietary choices

Table 3 shows the percentage of people who were aware of nutritional components during food choices in the intervention period. The labeled group had a significantly higher percentage of people who were aware of the nutritional balance of their overall diet than the unlabeled group by nearly 40% (*p* =.002). Furthermore, for each nutritional component, the labeled group had a significantly higher proportion of people conscious of nutritional components than the unlabeled group in nutritional components other than dietary fiber (each *p* <.05). Furthermore, a significant increase was observed in proteins not listed on the label (*p* =.045). Among the nutritional components, the strongest effects of TLF labeling were for salt (Cramer’s *V* = 0.50) and total lipid (Cramer’s *V* = 0.49). No significant difference was observed for health awareness (*p* =.48).

**Table 3:** Percentage of people who were conscious of nutritional components when choosing meals for the week in the intervention period

	Unlabeled group	Labeled group	Cramer’s V	p-value
	(*n* = 35)	(*n* = 34)		
Whole nutrition	11 (31.4%)	24 (70.6%)	0.39	**0.002**
Calories	8 (22.9%)	19 (55.9%)	0.34	**0.007**
Proteins	8 (22.9%)	16 (47.1%)	0.25	**0.045**
Total lipids	5 (14.3%)	21 (61.8%)	0.49	**< 0.0001**
Sugar	1 (2.9%)	9 (26.5%)	0.34	**0.006**
Saturated fatty acids	1 (2.9%)	11 (32.4%)	0.39	**0.001**
Salt	2 (5.7%)	17 (50.0%)	0.50	**<0.0001**
Dietary fiber	3 (8.6%)	5 (14.7%)	0.10	0.48
Health awareness	16 (45.7%)	21 (61.8%)	0.16	0.23

### Effects of TLF label use on psychological stress

Whether the use of TLF labeling affects stress during food choices was assessed using DAS and VAS (Table 4). The results showed no significant differences in all endpoints between the groups.

**Table 4:** Comparison of changes in stress between baseline and intervention periods

Variables	Adjusted mean (95% CI)			
	Unlabeled group	Labeled group	LSMD	p-value
DASS				
Total	0.34 (− 1.49 to 2.18)	−0.03 (− 1.89 to 1.83)	0.37 (− 2.24 to 2.99)	0.78
Depression	0.08 (− 0.71 to 0.88)	0.01 (− 0.79 to 0.82)	0.07 (− 1.06 to 1.20)	0.90
Anxiety	−0.06 (− 0.54 to 0.42)	−0.26 (− 0.75 to 0.22)	0.21 (− 0.48 to 0.89)	0.55
Stress	0.32 (− 0.53 to 1.18)	0.19 (− 0.68 to 1.05)	0.14 (− 1.08 to 1.35)	0.82
VAS				
VAS1	−0.58 (− 1.38 to 0.21)	−0.30 (− 1.11 to 0.51)	−0.28 (− 1.42 to 0.85)	0.62
VAS2	−0.30 (− 1.11 to 0.51)	−0.16 (− 0.98 to 0.66)	−0.14 (− 1.30 to 1.01)	0.81
VAS3	0.07 (− 0.60 to 0.75)	0.29 (− 0.40 to 0.98)	−0.22 (− 1.18 to 0.75)	0.66

VAS1: Looking back over the past week, have you lived a regular life? VAS2: How stressful did you look back in the last week? VAS3: How much light did you spend in your mood throughout the last week?

### Questionnaire survey on TLF label at the end of the trial

In response to the question “Do you think that colored nutrition labels on the front improve health awareness?,” 97.1% of the participants in the nutrition label group and 100.0% in the unlabeled group answered that they think so. In response to the question “Do you want manufacturers to put color nutrition labeling on the front of food packaging?,” 91.3% of the participants responded that they like this. Additionally, in response to “Do you think that colored nutrition labels on the front are effective?,” 98.6% of the participants responded that they are effective (Table 5).

**Table 5:** Questionnaire survey on TLF label at the end of the trial

	Unlabeled group	Labeled group	Total	
	(*n* = 35)	(*n* = 34)	(*n* = 69)	*p*-value
Do you usually use the nutritional information label on the back of the package?	15 (42.9%)	23 (67.7%)	38 (55.1%)	0.05
Do you think it would be better to use a colored nutrition label on the front of the package in addition to the current nutrition label on the back?	34 (97.1%)	33 (97.1%)	67 (97.1%)	1.00
Do you think that colored nutrition labels on the front improve health awareness?	35 (100.0%)	33 (97.1%)	68 (98.6%)	0.49
Do you think that colored nutrition labels on the front are effective?	35 (100.0%)	33 (97.1%)	68 (98.6%)	0.49
Do you want manufacturers to put color nutrition labeling on the front of food packaging?	32 (91.4%)	31 (91.2%)	63 (91.3%)	1.00

## Discussion

In this study, 70 college students were randomly divided into two groups to assess whether they would choose a healthy diet using TLF labels through a controlled trial of food choices. The results showed that the labeled group chose a healthier diet than the unlabeled group throughout the week. The comparison of the total scores calculated by scoring the color of the nutrition labels showed that the nutrition label group showed significantly lower values than the unlabeled group. This indicated that the use of TLF labels increased the percentage of those who chose a healthier meal. The results also showed that they became aware of the nutritional content that was not displayed in addition to the nutritional content listed on TLF labels at the time of dietary choices. No stress was noted with the use of nutrition labels in this study. Although there have been reports evaluating the short-term usefulness of TLF labels, there have been no reports of interventional studies evaluating the effects of labels when nutrition labels are used continuously. This study investigated the change in scores on TLF labels when using nutritional labels at the time of food selection throughout the week and showed that the labeled group had lower scores than the unlabeled group. While it has been reported that 50% of the Japanese are becoming more health conscious due to the COVID-19 crisis, the health consciousness of young people in their 20s in Japan is lower than that of other generations, only about 35%. In this study, the effect was observed in youth with low health awareness, so other generations may benefit from nutrition labeling.

In the aggregated results based on the color of the nutrition label for food choices during the baseline and intervention periods, the diet choices of the two groups were similar during the baseline period. However, a significant difference in the proportion of nutrition labels was observed between the two groups during the intervention period. That is, 11% more patients in the labeled group chose a diet falling under the blue label, and 13% less often chose a diet falling under the red label. Previous overseas reports have shown that using nutrition labels at the time of food selection increases the proportion of dietary choices falling under the blue label and decreases the proportion of dietary choices falling under the red label. These results are similar to those observed in this study, suggesting that it may be beneficial to use nutrition labels when choosing a meal in Japan and other countries.

Regarding whether TLF labels affect awareness of nutritional components during food selection, the TLF-labeled group had a significantly higher percentage of individuals who were aware of nutritional aspects during food selection by nearly 40% than the TLF-unlabeled group. This time, four components, sugar, saturated fatty acids, total lipids, and calories, were labeled based on TLF labels in the UK. However, significant differences were also observed for the nutritional content of proteins not labeled on TLF labels. This use of TLF labels has been reported to increase the awareness of nutritional components not listed on the labels. Our findings indicate that the same benefits can be achieved even when used continuously. This suggested that the continuous use of TLF labels influenced the awareness of the consumption of healthy nutritional ingredients, regardless of whether nutritional ingredients were described or not. However, no significant results were obtained for dietary fiber. This may be because few of the meal image options contained dietary fiber, so they may have ceased to be conscious of it while repeatedly viewing images and making meal selections.

When examining whether the use of daily TLF labeling affected stress, the current survey found no significant differences between the groups. This suggests that the labeling of nutrition labels facilitates healthy dietary choices on a daily basis without creating psychological stress.

In a questionnaire-based survey on TLF labels, more than 90% of participants in both groups responded “I think TLF labels are helpful” and “I want to have TLF labels on the front of the food package.” Additionally, only 55% of the participants responded “I use the nutrient labeling that is usually listed on the back of packaging.” These results suggest that TLF labels on the front of food are more convenient and more understandable than the nutritional labels on the back, which are not used much in Japan [12]. The TLF labels are therefore suitable for the needs of consumers and are beneficial because they provide an immediate understanding of nutritional components and allow healthy dietary choices even without nutritional knowledge.

This study has some limitations that should be addressed in future research. Firstly, the study focused solely on the selection of dinner meals. In reality, dietary choices are made multiple times throughout the day, with previous research indicating that the impact of nutrition labels is most significant at lunchtime. Hence, a study conducted during lunch could potentially reveal a clearer effect of nutrition labels on food choices.

Secondly, although the participants made choices about meals, they did not actually consume the meals. While selecting meals from a menu with images is a common practice in Japan, mirroring typical dietary choice situations, the absence of actual consumption could influence the study’s findings. Therefore, caution must be exercised when interpreting the results.

Thirdly, the demographic makeup of the study participants, with a significant majority being female (92.8%), presents another limitation. A report suggests that women are more likely to use and be influenced by nutritional labels than men [45]. There is a possibility that the proportion of female participants was higher because women tend to have more interest in diet than men. Additionally, it is a fact that Japanese pharmacy departments have a high percentage of female students, approximately 70%, which may have contributed to the higher representation of women in our study. The high proportion of female participants in this study may have introduced a gender bias, affecting the generalizability of the results. Future studies should consider evaluating the impact of nutrition labels on both genders separately, ensuring a more comprehensive understanding of the effects of nutritional labeling and accounting for potential differences in how nutritional information influences meal choices across genders.

Fourthly, when considering the social desirability bias associated with balanced diets among university students, the use of self-reported data through online surveys raises additional concerns regarding the reliability and accuracy of participants’ answers about their behaviors and perceptions.

Finally, one significant limitation of our study is the use of images for meal selection without actual food consumption. This method, though reflective of the common Japanese practice of choosing meals from menu photographs, differs from Western countries where menus are often text-based. This cultural contrast suggests TLF labels might be more effective in a visual menu context like in Japan. Recognizing this, future studies should include actual consumption to provide a more comprehensive evaluation of nutritional labels’ impact across different cultural settings.

The study’s strength is that it conducted a double-blind randomized controlled trial to assess the effectiveness of 1-week TLF labeling in Japanese university students. This study showed that the use of TLF labels increased the percentage of people who were aware of healthy eating, indicating the effectiveness of using TLF labels continuously for Japanese university students. In our study, the use of Google Forms allowed us to monitor participants’ responses in real time, ensuring that all responses were submitted promptly within the same day without delay. Furthermore, due to the randomized allocation, we believe that the behavior and perception of participants between the two groups are balanced on average, which ensures comparability and lends credibility to the results obtained from our study. Further studies are needed to investigate the usefulness of TLF labels attached to actual meals as a trial and to examine the nutritional labels used worldwide other than the TLF labels used this time. It would also be useful to consider which nutrition labels are most effective and suitable for the Japanese, for example, Nutri-Score (France) [18, 46], Nutritional warning systems (Chile) [26, 27], and HSR (Australia) [25]. Additionally, conducting deeper investigations into how nutritional awareness varies among various demographics beyond university students would provide insights into the broader applicability of TLF labels. Furthermore, this study focuses on a short-term intervention (1 week). It would be interesting to see research on the long-term effects of TLF labeling on dietary choices, which could provide more insights into how these labels influence eating habits over extended periods.

Previous studies on food choices have shown that people who read nutrition labels on the back of food packages tend to have the intention to eat healthier diets [47]. However, this label is daily used only by a small portion of consumers [48]. Various FOPL interpretation systems, including nutrition labels other than the TLF label, have been reported to be associated with healthier food choices [49–55]. Therefore, it would be beneficial to consider using FOPL, such as the TLF label, to prevent lifestyle-related diseases through healthy dietary choices in Japan.

## Conclusion

The use of TLF labeling increased the percentage of people who opt for a continuously healthy diet throughout one week and raised awareness of nutritional components. This approach shows promise for promoting healthier choices and preventing lifestyle-related diseases in Japan. Future research should investigate its broader impact on diverse demographics. Policymakers are advised to consider these findings for public health strategies, potentially informing both national and global nutritional labeling practices.

## Data Availability

The data are not publicly available as all participants have not consented to the public disclosure of the data online. However, the data presented in this study are available from the corresponding author upon request.

## Abbreviations

FOP: Front of food packaging
FOPL: Front of Pack Food Labels
GDA: Guideline daily amount
LSMD: Least squares mean difference
TLF: Traffic Light Food
VAS: Visual Analog Scale
